# Effects of psychosocial factors on nonadherence to ART in Ganta, Nimba county, Liberia

**DOI:** 10.1186/s12981-022-00455-2

**Published:** 2022-06-25

**Authors:** Philomena J. Strother, Mathuros Tipayamongkholgul, Varakorn Kosaisevee, Nawarat Suwannapong

**Affiliations:** 1 Jackson F. Doe Memorial Regional Referral Hospital, (MOH), Tappita, Liberia; 2 Faculty of Public Health, Mahidol University, 420/1 Ratchawithee Road, Rajthewee, Bangkok, 10400 Thailand; 3 Department of Epidemiology, Faculty of Public Health, Mahidol University, 420/1 Ratchawithee Road, Rajthewee, Bangkok, 10400 Thailand

**Keywords:** HIV-related stigma, Social support, ART-nonadherence, Liberia

## Abstract

**Background:**

It has been widely noted that lifetime adherence to antiretroviral therapy (ART) is necessary for HIV treatment outcome; however, retention on ART among people living with HIV (PLWH) remains a great challenge to achieve the Global AIDS Strategy: End inequalities, End AIDS. Nonadherence to ART is one of the HIV care problem in Liberia despite the availability of free ART. Psychosocial factors, i.e., perceived stigma and social support likely contributed to nonadherence to ART. We investigated associations among clinical factors, psychosocial factors, and nonadherence to ART.

**Methods:**

A community-based cross-sectional study was conducted among 185 PLWH, age ≥ 18 years receiving ART in Ganta, Nimba county, Liberia at least 3 months. The structured questionnaire was used to collect data from April to May 2020. Associated factors of nonadherence to ART were identified using multivariable binary logistic regression, and the p-value < 0.05 was considered statistically significant.

**Results:**

Of 185 respondents, 62.2% showed nonadherence to ART. Females reported higher nonadherence compared with males (64.4% vs. 56.6%). Multivariable binary logistic regression revealed strong experiences of stigma (PORadj = 2.392, p-value = 0.018), poor information support (PORadj = 2.102, p-value = 0.026) increased prevalence of ART nonadherence among Liberian PLWH.

**Conclusions:**

The healthcare providers may apply interventions to reduce perceived stigma and to enhance continuous information provision in addition to support from health care providers and family members. An intensive monitoring of ART side effects is needed to be strengthened in particular among newly started ART patients.

## Introduction

The global burden of human immunodeficiency virus (HIV) has been greatly attributed to sub-Saharan Africa. More than 70% of HIV incidence and 50% of deaths among them were attributed to the region [1]. In the past decade, the number of HIV incidences and deaths in this region have been reduced by 44% and 20%, respectively, due to the great efforts on the 90-90-90 HIV treatment targets by the Joint United Nations Programme on HIV/AIDS (UNAIDS) [1, 2]. Although the improved situation of HIV/AIDS in sub-Saharan Africa has been observed, the progress of continuum testing and treatment services in western and central Africa are slow and far behind others in sub-Saharan Africa [3].

In western and central Africa, the incidence-prevalence ratio was 5.5% about twice higher than the benchmark for epidemic control of 3% [3]. Among people living with HIV (PLWH) in this region, only 64% knew their status, 50% were being treated, and 39% successfully suppressed viral load [4]. Liberia is located in western and central Africa where less than 40% of PLWH are being treated and less than 20% can suppress viral load [5], despite the national government providing free of charge antiretroviral treatment (ART) [5]. The situations highlighted inequality to access HIV services in Liberia and could constitute barriers to reach the new Global AIDS Strategy 2021–2026: End Inequalities, End AIDS, and the United Nations General Assembly Political Declaration on HIV and AIDS: Ending Inequalities and Getting on Track to End AIDS by 2030 [6].

The accessibility and coverage to ART has been well-improved worldwide, but retention on ART among PLWHs remains a great challenge to achieve the global targets [1–4]. It has been widely noted that lifetime adherence to ART is necessary for viral suppression and HIV treatment outcomes [6]. Viral suppression retention can reduce HIV-related mortality and prevent HIV transmission [6–8]. To remain on ART among PLWH has continued the significant problems worldwide [9], studies in the US found that 40–55% of PLWH showed ART nonadherence [10, 11], same as a report in Lao PDR [12]. Better situations were reported in Myanmar, Tanzania and Thailand where the prevalence of non-adherence was lower than 20% [13–15]. ART nonadherence reduces ART effectiveness in particular viral suppression, and clinical outcomes and causes antiretroviral drug resistance [6, 16].

HIV-related stigma has widely affected HIV prevention and control, as well as ART adherence among PLWH worldwide. HIV-related stigma likely reduced motivation to continue ART among PLWH [17–19]. A study in African countries found that HIV-related stigma induced isolation and hopelessness among PLWH, and eventually reduced motivation to strictly comply with ART [19]. Accepting lifetime ART requires social supports that can enhance self-confidence of PLWH, the sense of being loved, and motivation to maintain ART [19, 20].

HIV-related stigma in African countries is prevalent [21, 22]. A population-based survey in western and southern Africa found more than 50% of adults would not buy vegetables from PLWH, and more than 35% of adults would not allow HIV positive children to attend school with other children [21]. Likewise, a survey by the Department of Health in Liberia found 53% of adults would not buy vegetables from PLWH [4, 5]. Related studies found a large proportion of ART nonadherence among PLWH with inadequate HIV knowledge and HIV-related stigma [23, 24]. Although association between HIV-related stigma, social supports and ART adherence were widely presented [19, 20, 25], different social contexts and health systems can vary results [26]. Our study aimed to examine the prevalence and association of ART nonadherence, HIV-related stigma and social supports among PLWH in Liberia.

## Methods

### Study design and study settings

The community-based cross-sectional study was conducted among PLWH living in Nimba county, Republic of Liberia from April to May 2020. Nimba county is located in northeastern Liberia, approximately 201 miles from Monrovia, capital of Liberia. This study was conducted after granting the protocol approval by the Ethics Review Committee for Human Research, Faculty of Public Health, Mahidol University and the National Research Ethics Board of Liberia.

The study population was PLWH registered at Ganta United Methodist Hospital, the largest health facility, servicing at least 60% of the residents, and the only facility providing ART in the area. PLWH, residing in Ganta, Nimba county, aged 18 years and older, and registered for ART for at least three months were included. Of 445 eligible PLWH, 185 PLWH were randomly selected and asked for permission to visit respondents’ homes. After receiving permission, the research assistants visited the respondents, and the interviews began after respondents signed the consent forms. The face-to-face interviews were conducted by research assistants in private areas around the respondents’ premises. Five research assistants passed the virtual one-day-training course to ensure their ability to properly proceed with a face-to-face interview.

### Research tools

The structured questionnaire included four sections, i.e., personal characteristics, psychosocial-related factors, knowledge and beliefs, and ART nonadherence.

The personal characteristics section comprised nine items to query respondents about age, sex, occupation, marital status, education, religion, monthly income, and experiences with side effects of ART.

Psychosocial-related factors section included HIV-related stigma and social support. HIV-related stigma comprised three domains: (1) experienced stigma referring to an act of discrimination PLWH faced; (2) internalized stigma referring to a feeling of shame and self-blaming because of being HIV-positive; and (3) perceived stigma referred to a feeling of being devalued by others because of his or her HIV-positive status. Each domain had five items of three-point ratings scale: 0 = disagree, 1 undecided and 2 = agree. The 15 items were summed to create a composite score that ranged from 0 to 6 by domain. Higher scores indicated a greater level of stigma.

Social support comprised four domains: (1) emotional support referring to experience of receiving empathy, care, and love from others; (2) information support referring to experience of receiving advice, suggestions, and HIV related information from others; (3) instrumental support referring to the experience of receiving tangible aids and services from others and (4) appraisal support referring to the experience of receiving information useful to make decision on HIV treatment from others. Each domain had four items of three-point rating scale: 0 = never, 1 = sometimes and 2 = always. The 12 items were summed to create a composite score ranging from 0 to 8 by domain. Higher scores indicated a greater level of available social supports.

The knowledge and belief section comprised four items of yes–no questions for knowledge on HIV and ART and six items of three-point rating scales regarding questions for beliefs on HIV and ART. The sum scores were created for knowledge ranged from 0 to 4 and beliefs ranged from 6 to18.

The ART nonadherence was assessed using three criteria including self-reporting methods (ask the patient about missing pills), delayed taking pills and missed appointment for drug refill. Failure to meet one of the criteria over the past three months was considered nonadherence to ART.

The Cronbach’s Alpha coefficients were calculated to determine reliability of the questionnaire. Thirty PLWH formed a subsample from the eligible subjects and were interviewed. The Cronbach’s Alpha coefficients revealed optimal results of 0.671 for knowledge, 0.795 for beliefs, 0.871 for social supports, and 0.722 for stigma.

### Analysis

All composite variables were categorized based on criteria to three groups, i.e., poor/low/negative (score < 60%), moderate/neutral (score 60–79%), good/high/positive (score ≥ 80%). Number and percentage were used to describe categorical variables, and mean and standard deviation was used to describe continuous variables. Binary logistic regression was used to calculate the prevalence odds ratio (POR) of nonadherence to ART. All domains of stigma and social support were further analyzed using multivariable binary logistic regression adjusted for ART side effects and significant personal characteristic factors. Significant level was considered at < 0.05.

## Results

Of 185 PLWH, 62% showed nonadherence to ART. About 56.2% of the respondents were aged 40 years and older, and 24.8% married. Among all respondents, 71.4% were female, 64.3% unemployed and 62%had lower than high school education level. Ninety-eight percent of participants were Christian, and 39% reported experiencing ART side effects (Table 1).

**Table 1:** Nonadherence to art and participant’s characteristics (n = 185)

Factors	n	%
Nonadherence	115	62.2
Age group (years)		
< 30	19	10.3
30–39	62	33.5
40–49	48	25.9
≥ 50	56	30.3
Mean, SD (years): 43.5, 11.9 Min–Max: 22–72		
Sex		
Male	53	28.6
Female	132	71.4
Occupation		
Unemployed	119	64.3
Self-employed	44	23.8
Private employees	14	7.6
Government employees	8	4.3
Marital status		
Single	98	53.0
Married	46	24.8
Widowed/widower	24	13.0
Separated/divorced	17	9.2
Education level		
No formal education	44	23.7
Primary school	31	16.8
Secondary school	41	22.2
High school	63	34.1
Bachelor degree	6	3.2
Religion		
Christian	182	98.4
Others	3	1.6
Monthly income (Liberian dollar, LRD)		
2000–5000	40	21.6
5500–10,000	83	44.9
10,500–15000	35	18.9
> 15,000	27	14.6
Mean, SD (LRD): 10,780, 8738 Min–Max: 2000–62000 (LRD)		

USD = 200 LRD; Minimal wage/month = 5600 LRD

**Fig. 1:**
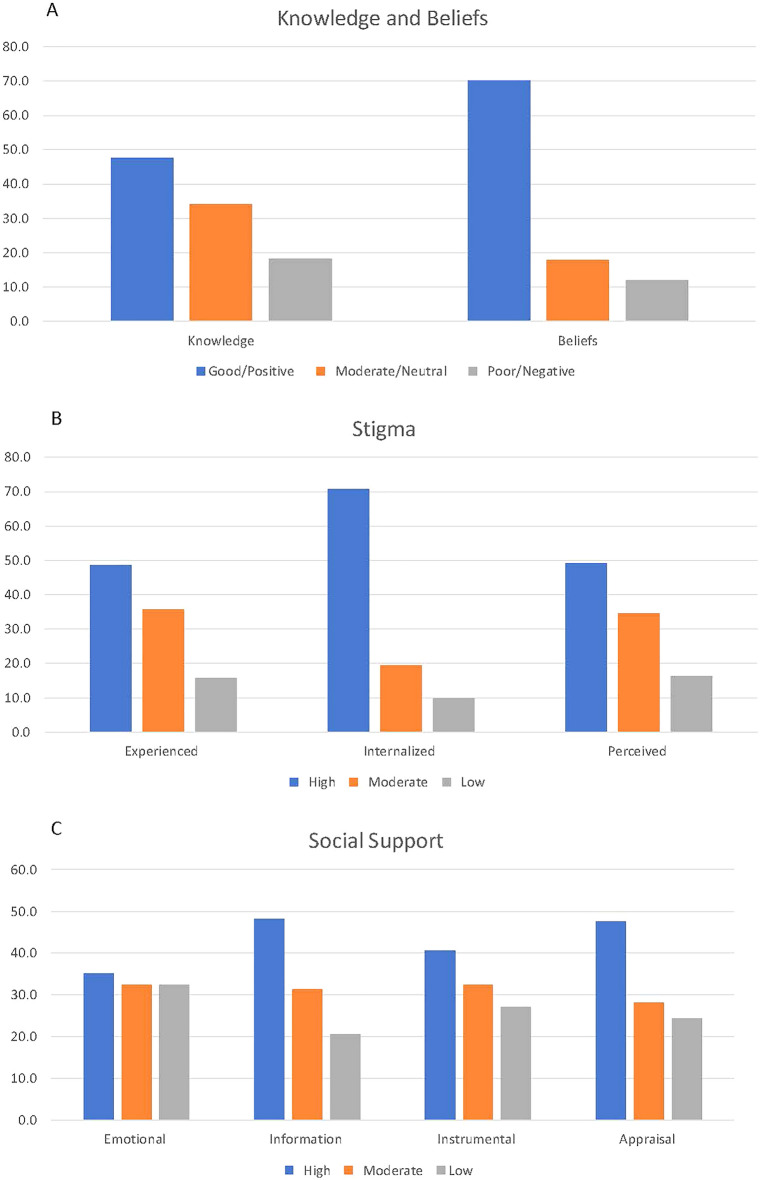
**A** Knowledge and belief level, **B** Stigma, and **C** Social support among PLWH

Although less than 50% of respondents exhibited good knowledge on HIV and ART level, 70% of respondents expressed positive beliefs about HIV and ART (Fig. 1A). About 70% of respondents had high levels of internalized stigma while almost 50% of participants had high levels of experienced and perceived stigma (Fig. 1B). Less than 50% of participants reported a high level of social support in all domains (Fig. 1C).

Binary logistic regression was used to quantify the magnitude of association between personal characteristics, knowledge and beliefs, domains of HIV-related stigma and domains of social supports. Due to small number of subjects of the subgroup in some factors, we collapsed some homogenous subgroups before analyzing binary logistic regression. Bivariate analysis revealed that widowed or divorced PLWH were more likely to be nonadherent than single respondents (POR = 2.424, 95% CI 1.071–5.486). PLWH experiencing side effects of ART were less likely to be nonadherent to ART than those in the experienced group (POR = 0.368, 95% CI 0.199–0.682) (Table 2).

**Table 2:** Association between personal characteristics and nonadherence to ART by bivariate analysis

Variable	Adherence	Non-adherence	POR	95% CI		*p*
	n (%)	n (%)		Lower	Upper	
Age group (years)						
22–39	36 (44.4)	45 (55.5)	Ref			
40–49	14 (29.2)	34 (70.8)	1.943	0.908	4.159	0.087
> 50	20 (35.7)	36 (64.3)	1.440	0.715	2.902	0.308
Sex						
Male	23 (43.4)	30 (56.6)	Ref			
Female	47 (35.6)	85 (64.4)	1.387	0.724	2.655	0.324
Education level						
No formal education	16 (36.4)	28 (63.6)	Ref			
Primary	11 (35.5)	20 (64.5)	1.039	0.398	2.709	0.938
Secondary	17 (41.5)	24 (58.5)	0.807	0.337	1.933	0.630
High school and above	26 (37.7)	43 (62.3)	0.945	0.432	2.069	0.888
Occupation						
Unemployed	51 (42.9)	68 (57.1)	Ref			
Self-employed	13 (29.5)	31 (70.5)	1.788	0.851	3.758	0.125
Monthly employees	8 (27.3)	16 (72.7)	2.000	0.731	5.470	0.177
Marital status						
Single	43 (43.9)	55 (56.1)	Ref			
Married	17 (37.0)	29 (63.0)	1.334	0.65	2.738	0.433
Widowed/divorced	10 (24.4)	31 (75.6)	2.424	1.071	5.486	0.034
Religion						
Christian	69 (37.9)	113 (62.2)	Ref			
Muslim	1 (33.3)	2 (66.7)	1.221	0.109	13.721	0.871
Monthly income (LRD)						
2000–5000	18 (45.0)	22 (55.0)	Ref			
5500–10,000	34 (41.0)	49 (59.0)	1.179	0.551	2.524	0.671
10,500–15,000	9 (25.7)	26 (74.3)	2.364	0.886	6.305	0.086
> 15,000	9 (33.3)	18 (66.7)	1.636	0.594	4.511	0.341
ART side effect						
No	32 (28.6)	80 (71.4)	Ref			
Yes	38 (52.1)	35 (47.9)	0.368	0.199	0.682	0.001
Knowledge level						
Good	36 (40.9)	52 (59.1)	Ref			
Moderate and poor	34 (35.1)	63 (64.9)	1.283	0.707	2.327	0.412
Beliefs toward ART						
Positive	51 (39.2)	79 (60.8)	Ref			
Neutral and Negative	19 (34.5)	36 (65.5)	1.223	0.634	2.362	0.548

*POR* prevalence odds ratio

Among PLWH, those receiving moderate and poor information support levels were more likely to be nonadherent to ART than those with good levels (POR = 2.170, 95% CI 1.184–3.977). Similarly, PLWH experiencing high stigma levels showed significantly higher nonadherence to ART compared with those experiencing low levels (POR = 2.123, 95% CI 1.156–3.906) (Table 3).

**Table 3:** Association among knowledge, beliefs, social support and perceive stigma and nonadherence to ART by bivariate analysis

Variable	Adherence	Non-adherence	POR	95% CI		*p*
	n (%)	n (%)		Lower	Upper	
Social support						
Emotional support						
High	28 (43.1)	37 (56.9)	Ref.	0.758	2.607	0.280
Moderate and low	42 (35.0)	78 (65.0)	1.405			
Information support						
High	42 (47.2)	47 (52.8)	Ref.			
Moderate and low	28 (29.2)	68 (70.8)	2.170	1.184	3.977	0.012
Instrumental support						
High	33 (44.0)	42 (56.0)	Ref.			
Moderate and low	37 (33.6)	73 (66.4)	1.550	0.848	2.835	0.155
Appraisal support						
High	35 (39.8)	53 (60.2)	Ref.			
Moderate and low	35 (36.1)	62 (63.9)	1.170	0.645	2.121	0.605
Stigma						
Experienced stigma						
High	26 (28.9)	64 (71.1)	2.123	1.156	3.906	0.015
Moderate and low	44 (46.3)	51 (53.7)	Ref.			
Internalized shame						
High	44 (33.6)	87 (66.4)	1.834	0.963	1.038	0.065
Moderate and low	26 (48.1)	28 (51.9)	Ref.			
Perceived stigma						
High	29 (31.9)	62 (68.1)	1.652	0.907	3.012	0.101
Moderate and low	41 (43.6)	53 (56.4)	Ref.			

*POR* prevalence odds ratio

All domains of stigma and social support were further analyzed using multivariable binary logistic regression adjusted for marital status and experience of ART side effect. The results found that PLWH with poor levels of information support and high levels of experiencing stigma were more likely to exhibit nonadherence than those with good levels of social support and low levels of experiencing stigma (Adjusted POR = 2.102, 95% CI 1.092–4.046 and Adjusted POR = 2.392, 95% CI 1.161–4.914, respectively) (Table 4).

**Table 4:** Adjusted prevalence odds ratio for nonadherence to ART among PLWH

Variable	Adherence	Non-adherence	Adjusted POR	95% CI		*p*
	n (%)	n (%)		Lower	Upper	
Information support						
High	42 (47.2)	47 (52.8)	Ref.			
Moderate and low	28 (29.2)	68 (70.8)	2.102	1.092	4.046	0.026
Experienced stigma						
High	26 (28.9)	64 (71.1)	2.392	1.161	4.914	0.018
Moderate and low	44 (46.3)	51 (53.7)	Ref.			

Adjusted for marital status and experienced antiretroviral drug side effects, *Adjusted POR* Adjusted prevalence odds ratio

p-value < 0.05 is considered statistically significant

## Discussion

Our study emphasized the public health needs on mitigating HIV-related stigma and enhancing social supports to decrease ART nonadherence in Ganta, Nimba county, Liberia. After adjusting for personal factors and side effects of ART, we found that high levels of experienced stigma and poor levels of information support increased ART nonadherence two folds. On the other hand, social support could promote ART adherence. It has been widely noted that nonadherence causes poor clinical outcomes among PLWH [26] and drug resistance [8, 16].

Prevalence of nonadherence in this study was higher than that of studies in Lao PDR, Myanmar, Tanzania, and Thailand [11–14] but similar to studies in the US [10, 11]. Being forgetful or being late taking pills was commonly reported among PLWH. Daily HIV treatment pushed PLWH to accept life-long medication and to adapt life styles, and socialization patterns [19]. PLWH may feel healthy and want to resume their social lifestyle and normal life activities, but ART likely interfered with their sense of normal life [28].

Poor ART adherence among PLWH experiencing stigma in our study was similar to that of studies in Africa [18, 19, 22], Asia [12, 13, 15, 25], the Caribbean [27], and the US [10]. Dudley defined stigma as “stereotypes or negative views attributed to a person or groups of people when their characteristics or behaviors are viewed as different from or inferior to societal norms” [29]. PLWH, having or acquiring stigma, believe that others will reject and devalue them because of their HIV status. Stigma can reduce one’s self-esteem and lessen one’s value in life. Due to such psychological conditions, PLWH commonly disengage from social activities and decrease motivation to HIV care [9, 10, 19, 20]. Although internalized stigma did not present any association with ART nonadherence, our study found 70% of PLWH reported internalized stigma. Internalized stigma is characterized by feelings of shame, guilt and worthlessness and related to negative stereotypes in society. A related study emphasized that public stigma leads to internalized stigma [30]. The high prevalence of internalized stigma in our study may likely have resulted from PLWH experiencing stigma from the community.

This present study found higher ART nonadherence among those perceiving lower information supports twofold. The findings of social supports as a protective factor of ART nonadherence were reported in Tanzania [14] and Zimbabwe [20] but not in Myanmar [13], Thailand [15] and the US [10]. Different findings of social supports and ART nonadherence varied across countries because social supports strongly rely on social norms and structures [31]. It has been noted in a systematic review and studies in subSaharan Africa that social support can moderate the harmful effects of HIV-related stigma and minimize life stress [18, 20, 32].

## Conclusion

This study revealed high prevalence of ART nonadherence among PLWH in Ganta, Liberia. Association between social supports and experienced stigma with ART nonadherence highlighted the needs of interventions to reduce stigma and programs to provide social support. Collaborating among family members, health care providers and PLWH needs to be developed to decrease HIV-related stigma, increase social support and improve ART adherence among PLWH.

## Data Availability

The datasets generated and analyzed during the current study are not publicly available because they constitute an excerpt of research in progress but are available from the corresponding author on reasonable request.

## Code availability

Code availability is not applicable.
